# A Novel Thyrotropin-Releasing Hormone Receptor Missense Mutation (P81R) in Central Congenital Hypothyroidism

**DOI:** 10.1210/jc.2015-3916

**Published:** 2016-01-06

**Authors:** O. Koulouri, A. K. Nicholas, E. Schoenmakers, J. Mokrosinski, F. Lane, T. Cole, J. Kirk, I. S. Farooqi, V. K. Chatterjee, M. Gurnell, N. Schoenmakers

**Affiliations:** Metabolic Research Laboratories (O.K., A.K.N., E.S., J.M., I.S.F., V.K.C., M.G., N.S.), Wellcome Trust-Medical Research Council Institute of Metabolic Science, University of Cambridge and National Institute for Health Research, Cambridge Biomedical Research Centre, Addenbrooke's Hospital, Cambridge CB2 0QQ, United Kingdom; West Midlands Regional Genetics Service (F.L., T.C.), Birmingham Women's Hospital NHS Foundation Trust, Birmingham B15 2TG, United Kingdom; and Department of Endocrinology (J.K.), Birmingham Children's Hospital, Birmingham B4 6NH, United Kingdom

## Abstract

**Context:**

Isolated central congenital hypothyroidism (CCH) is rare and evades diagnosis on TSH-based congenital hypothyroidism (CH) screening programs in the United Kingdom. Accordingly, genetic ascertainment facilitates diagnosis and treatment of familial cases. Recognized causes include TSH β subunit (*TSHB*) and Ig superfamily member 1 (*IGSF1*) mutations, with only two previous reports of biallelic, highly disruptive mutations in the TRH receptor (*TRHR*) gene.

**Case Description:**

A female infant presenting with prolonged neonatal jaundice was found to have isolated CCH, with TSH of 2.2 mU/L (Reference range, 0.4–3.5) and free T_4_ of 7.9 pmol/L (0.61 ng/dL) (Reference range, 10.7–21.8 pmol/L). Because *TSHB* or *IGSF1* mutations are usually associated with profound or X-linked CCH, *TRHR* was sequenced, and a homozygous mutation (p.P81R) was identified, substituting arginine for a highly conserved proline residue in transmembrane helix 2. Functional studies demonstrated normal cell membrane expression and localization of the mutant TRHR; however, its ability to bind radio-labelled TRH and signal via Gqα was markedly impaired, likely due to structural distortion of transmembrane helix 2.

**Conclusions:**

Two previously reported biallelic, highly disruptive (nonsense; R17*, in-frame deletion and single amino acid substitution; p.[S115-T117del; A118T]) *TRHR* mutations have been associated with CCH; however, we describe the first deleterious, missense *TRHR* defect associated with this phenotype. Importantly, the location of the mutated amino acid (proline 81) highlights the functional importance of the second transmembrane helix in mediating hormone binding and receptor activation. Future identification of other naturally occurring *TRHR* mutations will likely offer important insights into the molecular basis of ligand binding and activation of TRHR, which are still poorly understood.

Isolated central congenital hypothyroidism (CCH) occurs when there is inadequate TSH-mediated stimulation of the thyroid gland. To date, genetic disorders affecting TSH biosynthesis have been restricted to mutations in three genes, namely *TSHB*, *TRHR*, and *IGSF1* (1). TSH is inappropriately normal or subnormal, precluding diagnosis on the UK TSH-based congenital hypothyroidism (CH) screening program, and delayed initiation of treatment with T_4_ may compromise growth and neurological development. Biallelic *TRHR* mutations have hitherto been described in only two unrelated kindreds in which three affected individuals exhibited CCH and absent TSH and prolactin responses to exogenous TRH. In the first kindred, one affected individual was compound heterozygous for a paternally inherited nonsense mutation (R17*) and a complex combination of mutations on the maternal allele comprising an in-frame deletion (S115, I116, and T117) with one amino acid substitution (p.A118T). In the second kindred, two affected individuals were homozygous for the R17* mutation (2, 3).

TRH receptor (TRHR) is a G protein-coupled plasma membrane receptor (GPCR) belonging to the largest subclass (family A, rhodopsin-like 7 transmembrane domain receptors). Hypothalamic TRH signaling via TRHR primarily involves mobilization of intracellular calcium and activation of protein kinase C through a Gq/11-dependent pathway (4, 5). TRH both mediates transcriptional activation of target genes, eg, TSH α-subunit (*CGA*) and β-subunit (*TSHB*), and exerts post-translational effects, eg, conjugation, glycosylation, and secretion of the α-subunit/TSHβ heterodimer (TSH) in the anterior pituitary (6).

Here, we describe a female Pakistani infant diagnosed with CCH in the neonatal period. Parental consanguinity favored recessive inheritance; thus, *TRHR* was the most likely causative gene given her moderate biochemical defect. Sequence analysis revealed a homozygous *TRHR* missense mutation (p.P81R), and molecular studies confirmed impaired ligand binding and transcriptional activation by the mutant receptor.

## Patient and Methods

All investigations were ethically approved and/or clinically indicated, being undertaken with patient or parental consent.

### Biochemical measurements

Hormone measurements were made using local automated assays (Supplemental Data).

### Molecular genetic studies

Sanger sequencing of *TRHR*, *TSHB*, and *IGSF1* coding exons was performed after PCR amplification of genomic DNA using specific primers (Supplemental Data).

### Molecular modeling

The TRHR structural model was generated by homology modeling using the PHYRE server and MacPymol to generate figures.

### Construction of wild-type and mutant TRHR cDNA expression vectors

Site-directed mutagenesis was used to generate P81R TRHR cDNA (Supplemental Data). Wild-type (WT) and P81R TRHR cDNAs were cloned into the eukaryotic expression vectors pcDNA3 and pEGFP-N3 (Clontech) upstream of FLAG and green fluorescent protein (GFP) epitope tags, respectively.

### Transient transfection

HEK293 cells were transiently transfected using Lipofectamine 2000 according to the manufacturer's instructions and cultured in DMEM supplemented with 10% FBS and 1% PSF on glass coverslips for cell localization assays, 96-well plates for functional assays, or 10-cm dishes for immunoprecipitation assays.

### Cell localization

HEK293 cells transfected with TRHR-GFP constructs (50 ng/coverslip) were incubated with CellMask Deep Red Plasma Membrane Stain and DAPI nuclear stain (Life Technologies) and analyzed using a Zeiss LSM 510 META confocal microscope, and images were processed with ZEN software (Zeiss).

### TSHα promoter activation

HEK293 cells cotransfected with TRHR-FLAG constructs (10 ng/well), TSHα-LUC reporter (150 ng/well) (7), and internal control plasmid BOS-β-galactosidase (30 ng/well) were incubated for 24 hours with growth medium containing TRH (0–100 nm) before measurement of luciferase and β-galactosidase activity.

### Gq signaling

TRHR signaling through Gqα protein was monitored using a secondary messenger inositol phosphate accumulation assay adapted from a published protocol (Supplemental Data).

### Radioligand binding

Receptor binding of [^3^H][3Me-His^2^]TRH was performed according to an adaptation of a published protocol (Supplemental Data).

### Immunoprecipitation

HEK293 cells transfected with TRHR-FLAG constructs (10 μg/dish) were lysed (RIPA buffer) after 24 hours, immunoprecipitated with anti-FLAG affinity gel (F3165; Sigma), and analyzed by Western blotting (AntiFlag, F3165, Sigma; β-actin, Ab8227, Abcam).

## Results

### Clinical and biochemical features

**Figure 1.**
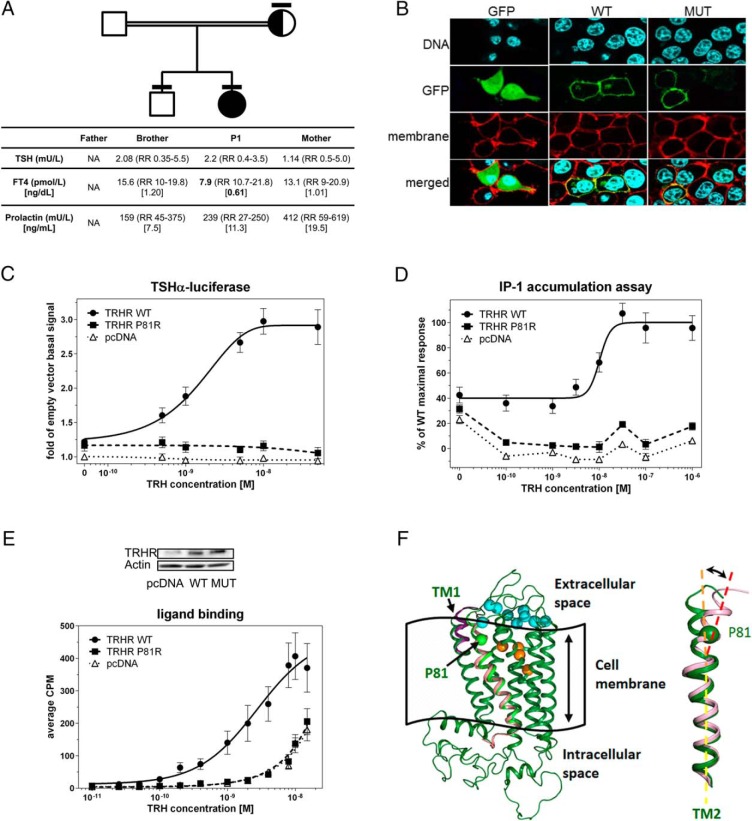
A, Central hypothyroidism segregates with homozygosity for the TRHR P81R mutation. Family pedigree showing the following genotypes: white, WT allele; black, mutant allele; black/white, heterozygous, with bars denoting individuals who have been genotyped. Biochemistry is tabulated at bottom of panel. NA, not available. B, WT and P81R TRHR both display similar plasma membrane localization. HEK293 cells were transfected with GFP, or GFP-tagged WT, or P81R TRHR. Nuclei (blue), plasma membrane (red), and GFP fusion (green) were visualized by immunofluorescence, and a composite merged image was generated. C, P81R mutant TRHR is unable to mediate ligand-dependent transactivation. HEK293 cells were transfected with empty vector (pcDNA), or FLAG-tagged WT, or P81R TRHR, together with the TRH-responsive TSHα-TKLUC reporter in an assay of TRH-dependent transactivation. BOS-β-galactosidase was used as an internal control. Data shown are normalized to maximal reporter gene activation with empty vector and represent the mean and SEM of at least 10 independent experiments. D, TRHR signaling via the Gqα-regulated cascade is impaired by the P81R mutation. HEK293 cells were transfected with WT, P81R, and empty (pcDNA) expression vectors and stimulated with increasing concentrations of TRH. TRHR signaling via the Gqα-regulated cascade was assessed by measuring accumulation of radio-labeled inositol monophosphate (IP-1) in the presence of Li^2+^, inhibiting its further dephosphorylation. IP-1 was detected with scintillation proximity assay beads with selective affinity for this secondary messenger. Data shown are normalized to maximal WT response to agonist stimulation and represent the mean and SEM of three independent experiments. E, Binding of TRH to P81R TRHR is markedly impaired. HEK293 cells were transfected with either empty vector (pcDNA), WT, or P81R TRHR and were incubated with increasing amounts of [^3^H][3Me-His^2^]TRH for 2 hours. A Western blot (inset) from immunoprecipitated samples confirmed expression of both WT and P81R TRHR. CPM, counts per minute. F, Molecular modeling of TRHR showing the position of P81 in TM2. The left panel shows TRHR positioned in the cell membrane. Amino acids from the surface ligand pocket are shown in cyan, and those of the main ligand binding cavity are shown in orange. Superimposed in pink is the position of TM2 containing the P81R mutation and in purple the position of TM1 when the mutation is present. The right panel shows a superimposition of WT (green) and mutant (pink) TM2 models, with the position of P81R identified, which is predicted to bring about a change in helix structure.

Our patient, the daughter of Pakistani parents who were first cousins, was born at 38 weeks of gestation by caesarean section due to placenta previa. She presented at age 19 days with neonatal jaundice due to isolated hyperbilirubinemia with bilirubin 174 μmol/L (10.2 ng/dL), (Reference range (RR), 0–120 μmol/L), for which she had no identifiable risk factors. The jaundice resolved spontaneously; however, despite a normal neonatal screening heel prick TSH (1 mU/L), she underwent further endocrine evaluation that revealed CCH with TSH of 2.2 mU/L (RR, 0.4–3.5) and free T_4_ of 7.9 pmol/L (0.61 ng/dL) (RR, 10.7–21.8 pmol/L). Basal pituitary function was otherwise normal: prolactin, 239 mU/L (11.3 ng/mL) (RR, 27–540 mU/L); cortisol, 220 nmol/L (8 μg/dL) (midday); and IGF-1, 6.7 nmol/L (51.3 ng/mL) (RR, 2.6–17 nmol/L). Pituitary magnetic resonance imaging was unremarkable. Her birth weight was 2.81 kg (25th centile), and she grew and developed normally before the initiation of levothyroxine treatment at 2 months of age. Currently aged 4.2 years, her growth and neurological development have remained normal (height SDS, +0.27; weight SDS, +2.24; age, 3.8 years). Her mother and brother were euthyroid (Figure 1A); her father declined investigation.

### Molecular genetic studies

*TRHR* sequencing revealed a homozygous single nucleotide substitution (c.242C>G) corresponding to a proline to arginine change at codon 81 (p.P81R), which is absent from normal genome datasets (dbSNP; Exome Aggregation Consortium [ExAC], Cambridge, MA; http://exac.broadinstitute.org, October 2015). Her mother and brother were heterozygous and WT, respectively (Figure 1A).

### Cellular localization

Although the P81R mutation occurs in transmembrane helix 2 (TM2) of TRHR, the P81R and WT TRHR-GFP fusion proteins demonstrated similar plasma membrane expression (Figure 1B).

### TRHR signaling

Transient transfection assays were used to interrogate: 1) transactivation of the TSH α-subunit promoter region, using a luciferase reporter construct; and 2) TRHR-coupled Gqα signaling by measuring accumulation of intracellular inositol phosphate. In both assays, P81R was nonfunctional compared with WT TRHR (Figure 1, C and D).

### Ligand binding

A ligand binding assay using the high-affinity ligand [^3^H][3Me-His^2^]TRH confirmed absent ligand binding by P81R TRHR, with normal binding to the WT TRHR (Figure 1E).

## Discussion

In two previously reported children with biallelic *TRHR* mutations, associated clinical manifestations were mild (growth retardation, delayed bone age) despite biochemical evidence of CCH, with T_4_ levels ranging from 40 to 88% of the lower limit of normal. Some bioactive TSH was produced, as evidenced by rising T_4_ levels after T_4_ withdrawal, and there was ostensibly no attributable neurological deficit despite treatment initiation at ages 9 and 11 years, suggesting sufficient childhood thyroid hormone production to prevent severe developmental delay. However, T_4_ replacement did improve growth and quality of life in these individuals (2, 3). Although TRHR is expressed on lactotrophs and mediates prolactin secretion in response to exogenous TRH, a female homozygote for p.R17* *TRHR* underwent two pregnancies and lactated normally, suggesting that TRHR is not obligatory for these functions in humans (3).

Our patient exhibits a *TRHR* missense mutation, which we presume to be homozygous because parental consanguinity would favor an autosomal recessive mode of inheritance and her mother is heterozygous. Unfortunately, her father declined genetic or biochemical evaluation, and thus we cannot exclude maternal isodisomy or a paternal deletion involving *TRHR*. Biochemical and radiological features in our patient (T_4_ levels 74% of the lower limit of normal, detectable TSH, normal basal prolactin, and normal pituitary magnetic resonance imaging) are concordant with previous cases and contrast with the profound hypothyroidism typically seen in children with *TSHB* mutations. Our patient's age (30 months) precluded cessation of T_4_ for TRH testing (2, 3).

The molecular mechanisms of TRH binding and TRHR activation remain poorly understood (8, 9). P81 has no known direct role in TRH binding but underlies a proline kink in TM2, which is conserved in 80% of GPCRs (Supplemental Figure 1). This is thought to influence ligand binding and signal transduction via an underlying hydrogen bond network, although its specific role in individual GPCRs may depend on the surrounding amino acids; mutation to alanine in CCR5 and AT1 receptors also results in impaired receptor activation (9). Our data support a critical functional role of P81 for ligand binding and thus receptor activation. We hypothesize that the P81R mutation alters the morphology of the proline kink in TM2, thus potentially affecting the positioning of TM1 and the extracellular loops and thereby altering the surface binding pocket (Figure 1F). Additional mutagenesis experiments are required to exclude an independent role of P81 in TRHR signal transduction.

This is the first report of a naturally occurring homozygous *TRHR* missense mutation, with similar clinical features to previous TRHR mutation cases. Despite the relatively mild reported sequelae of CCH due to TRHR mutations, T_4_ replacement ameliorates growth retardation and improves quality of life; additionally, detailed neuropsychological assessment (not undertaken in published cases) could plausibly delineate subtle neurological benefits (2, 3). Because these patients evade diagnosis in countries such as the United Kingdom that rely on a TSH-based CH screening program, genetic ascertainment is crucial in enabling early detection and treatment. Alternatively, inclusion of T_4_ measurement in the neonatal CH screening protocol (eg, in The Netherlands) enables early diagnosis of CCH, including cases with life-threatening additional pituitary hormone deficiencies. Identification of novel *TRHR* point mutations in isolated CCH cases may provide further molecular insights into TRHR function.
